# Microwave ablation for lymph node metastasis in thyroid cancer: the impact of lymph node diameter

**DOI:** 10.3389/fendo.2024.1430693

**Published:** 2024-08-06

**Authors:** Xiaoyi Xiao, Xi Chen, Jingwei Li, Pei Li, Yun Zhu

**Affiliations:** ^1^ Department of Radiology, The Third Xiangya Hospital, Central South University, Changsha, Hunan, China; ^2^ School of Nursing, The Hong Kong Polytechnic University, Hong Kong, Hong Kong SAR, China; ^3^ Joint Research Centre for Primary Health Care, The Hong Kong Polytechnic University, Hong Kong, Hong Kong SAR, China; ^4^ Department of Breast and Thyroid Surgery, The Third Xiangya Hospital, Central South University, Changsa, China; ^5^ Department of Ultrasound, The First Hospital of Hunan University of Chinese Medicine, Hunan University of Chinese Medicine, Changsha, Hunan, China

**Keywords:** microwave ablation, lymph node, papillary thyroid carcinoma, ultrasound, metastasis

## Abstract

**Objectives:**

To explore the impact of lymph node diameter on the efficacy and safety of ultrasound-guided microwave ablation (MWA) in the treatment of cervical metastatic lymph nodes (CMLNs) from thyroid cancer.

**Methods:**

A total of 32 patients with 58 CMLNs from thyroid cancer underwent ultrasound-guided MWA and were included in the retrospective study. Patients were divided into three groups based on the mean largest diameter of the CMLNs: Group A (diameter ≤10mm), Group B (10mm < diameter ≤20mm), and Group C (diameter >20mm). The research involved comparing changes in cervical metastatic lymph nodes and serum thyroglobulin (sTg) levels, as well as the incidence of complications, before and after microwave ablation across three groups of patients.

**Results:**

The technical success rate of this study was 100% (32/32), and they showed no major complications. Compared with measurements taken before MWA, the mean largest diameter and volume of CMLNs, as well as the sTg level, showed significant reductions (*p <*0.05) at the last follow-up in all three patient groups. Group A and B exhibited higher lymph node volume reduction rates and complete disappearance rates compared to Group C. However, the recurrence rate in the three groups were in the following order: Group C > Group B > Group A. The occurrence rate of mild complications was Group A > Group C > Group B.

**Conclusion:**

MWA is a safe and effective method for treating CMLNs, with advantages for localized nodes but limitations for larger ones. Careful consideration and personalized plans are advised, based on comprehensive evidence assessment.

## Introduction

Thyroid cancer is one of the most common endocrine malignant tumors globally, and its incidence has been rapidly increasing in recent years (1, 2). Papillary thyroid carcinoma (PTC) is the most common histological type, with a high cure rate. The 10-year survival rate exceeds 91%, and the 15-year survival rate exceeds 87% (3, 4). However, after surgical resection, 20-90% of PTC patients may develop cervical lymph node metastasis (CMLN), which is crucial for prognosis (5, 6). Currently, conventional treatments for CMLN from PTC include surgical resection, radioiodine ablation, and systemic therapy (7–9). However, repeat surgery carries a high risk of complications due to fibrosis and scar formation from previous surgeries (10, 11). Therefore, some non-invasive alternative techniques have been proposed, such as thermal ablation (12).

In recent years, ultrasound-guided microwave ablation (WMA) has gained significant attention as a thermal ablation therapy with good efficacy and safety in the management of CMLN from PTC (13, 14). The European Thyroid Association guidelines recommend it as one of the alternative surgical options for CMLN (15). Furthermore, as the size of the lymph nodes is crucial in treatment selection, the guidelines suggest: 1) CMLN smaller than 10mm may be considered for active surveillance (AS). However, some patients may eventually seek intervention due to factors such as psychological stress, leading to treatments like microwave ablation (16). 2) CMLN sized between 10-20mm may be considered for thermal ablation. Additionally, in clinical practice, many patients with CMLN larger than 20mm choose microwave ablation treatment out of concern for the risks of complications during repeat surgery.

Currently, there is insufficient direct evidence to explore the relationship between the efficacy and safety of MWA and CMLNs. Therefore, further investigation into the impact of CMLN diameter on the effectiveness and safety of ultrasound-guided MWA treatment is crucial. This research will provide important insights to optimize the treatment strategies for CMLN from PTC.

## Methods

### Study population

This study included a total of 32 patients with 58 CMLNs from PTC who underwent MWA treatment at The First Hospital of Hunan University of Chinese Medicine from December 2021 to December 2022. The study was divided into three groups: Group A with the mean largest CMLN diameter ≤10mm, Group B with the mean largest diameter greater than 10mm and less than or equal to 20mm, and Group C with the mean largest diameter greater than 20mm. Inclusion criteria were: (1) patients who underwent total thyroidectomy for PTC followed by at least one neck lymph node dissection; (2) patients continuously taking levothyroxine postoperatively to suppress serum thyroid-stimulating hormone; (3) patients with contraindications to surgery or refusal of surgical excision of CMLNs; (4) CMLNs detected by ultrasound (US) and diagnosed as PTC metastases using fine-needle aspiration (FNA) or washout thyroglobulin (Tg) concentration in FNA; (5) follow-up for at least 12 months after ablation. Exclusion criteria were: (1) children or pregnant women; (2) patients with distant metastases; (3) patients with severe bleeding tendencies; (4) patients with contraindications to the use of ultrasound contrast agents. This retrospective study has been approved by the Institutional Review Board of the First Hospital of Hunan University of Chinese Medicine. Due to the retrospective nature of the study, the requirement for informed consent was waived. All patients signed a written informed consent form for treatment before undergoing MWA.

### Equipment and methods

The MyLab Twice color Doppler ultrasound system (Esaote, Italy) utilized high-resolution linear probes (6-12 MHz) to monitor and guide various procedures, including cytological or histological examinations, pre-ablation assessments, ablation therapy, and follow-up. Additionally, the system was equipped with contrast-enhanced ultrasound imaging technology to improve diagnostic accuracy and provide better medical care for patients.

The KY-2000 2450 MHz microwave system (KY-2000, Kangyou Medical, Nanjing, China) manufactured by Nanjing Kangyou Medical was employed for microwave ablation (MWA). This system was specifically modified for the ablation of superficial organs. It consisted of a 16-gauge, Teflon-coated, internal-cooled microwave antenna with a 3-mm active tip and a 10-cm shaft.

### Pre-MWA assessment

Prior to microwave ablation (MWA), all patients underwent a detailed ultrasound examination. During this examination, we documented the location, size, blood flow, and surrounding characteristics of the cervical lymph nodes (LNs). The volumes of the lymph nodes were calculated using the formula V = πabc/6, where V represents volume, and a, b, and c represent the largest diameter and the two remaining perpendicular diameters, respectively. Additionally, contrast-enhanced ultrasound (CEUS) was used to assess the perfusion of the lymph nodes, and Tg testing was conducted before MWA.

### Procedure of MWA

**Figure 1:**
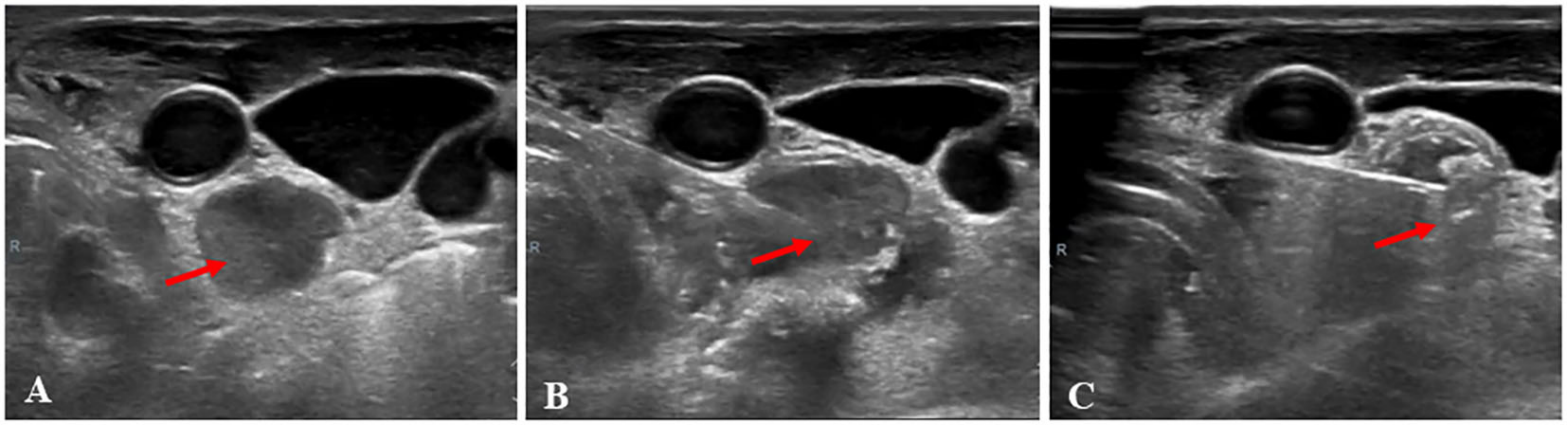
A 48-year-old woman, who had a total thyroidectomy three years ago due to papillary thyroid cancer, received microwave ablation (MWA) for cervical metastatic lymph nodes. **(A)**, Prior to MWA treatment, B-mode ultrasonography (US) showed a hypoechoic LN (arrow); **(B)**, The LN was punctured with a MWA needle (arrow); **(C)** During MWA, a hyperechoic pattern (arrow) was observed in the LN.

The MWA procedure was conducted under ultrasound guidance by a doctor with five years of experience in MWA. Patients were positioned in the supine position, and their necks were fully exposed. Before the procedure, local infiltration anesthesia with 1% lidocaine was administered, followed by an injection of normal saline around the lymph nodes such that the lymph nodes were moved to the safe side to protect nerves, blood vessels, and other important organs from burning (17). A 16 G sharp needle, 4 cm in length, was used to puncture the skin, and the antenna was inserted into the target lymph node via the tunnel that appeared after the needle was removed. In cases where insertion of the antenna was challenging due to small lymph nodes, the “ablating puncture” technique was employed, turning on the ablated mode after the antenna needle reached the surface of the lesion, followed by insertion into the lesion. For small lesions, fixed ablation was sufficient, while the “moving-shot” technique was applied for some larger lesions. The output power started at 10w or 15 w for small LN and gradually increased in steps to 30 w. The selection of the moving-shot, pull-back, or fixed applicator technique depends on the tumor’s specific characteristics (18). An ultrasound was used to observe a hyperechoic area during the ablation process, and ablation was terminated once the hyperechoic area covered the LN entirely (14). Afterward, CEUS was used to assess the efficacy of the ablation. If the contrast agent remained within the LN, the perfusion area was re-ablated. The MWA procedure was considered completed if no contrast agent was present in the LN. All patients in this study were hospitalized for the MWA. Patients were observed for two hours after MWA to monitor for any potential complications, such as bleeding, hoarseness, or skin burns. If a patient reports pain above 5 on the Visual Analogue Scale (VAS) after surgery, oral painkillers like ibuprofen are provided. The pre-ablation B-mode US and ablation process are depicted in **Figure 1**.

### Follow-up

Following MWA, cervical ultrasound was conducted at 1, 3, 6, and 12 months, followed by every six months thereafter. We documented the size of the lesions and calculated the volume and volume reduction rate (VRR) of the ablated LNs using the VRR equation: VRR (%) = ([initial volume – final volume] × 100%)/initial volume. At the three-month mark, all patients underwent another thyroglobulin test. For ablation areas, routine cervical B-mode ultrasound is performed, and CEUS and FNA are performed if metastasis or recurrence of the target LN is suspected. To rule out distant metastasis, computed tomography (CT) or ultrasound (US) examination was conducted every six months after MWA.

### Statistical analysis

Descriptive data are expressed as mean ± standard deviation. Statistical analysis was performed using R software (version 4.2.3, R Foundation for Statistical Computing, Vienna, Austria). To compare the measurements of cervical metastatic lymph nodes before and after microwave ablation (MWA), Shapiro-Wilk normality test was performed on the data. If the distribution is normal, a paired T-test is used; If not, the Wilcoxon signed rank test is used. Next, to evaluate the differences in Volume Reduction Rate (VRR) between the three groups, we first evaluated the Shapiro-Wilk normality Test and Levene’s test for homogeneity of variance of the data. If all are satisfied, One-way ANOVA statistical analysis is selected. If neither is satisfied, Kruskal-Wallis test is used for comparison. Chi-square test was used to compare the difference in complete disappearance rates among the three groups. Graphical representation using GraphPad Prism software 8.0 for Windows (GraphPad software, San Diego, California, USA). A P-value of less than 0.05 was considered statistically significant.

## Results

### Clinical and lymph node characteristics of participants

**Table 1** summarizes the demographic baseline data and lymph node characteristics of patients before MWA. The study included patients from three groups: Group A with 10 individuals, including 1 male and 9 females, with an average age of 42.00 ± 3.26 years; Group B with 14 individuals, including 3 males and 11 females, with an average age of 42.40 ± 7.66 years; Group C with 8 individuals, including 1 male and 7 females, with an average age of 43.11 ± 7.97 years. In the study population, the distribution of the primary tumor location was as follows: Group A had 7 cases on the right side of the neck and 3 cases on the left side of the neck; Group B had 9 cases on the right side of the neck and 5 cases on the left side of the neck; Group C had 2 cases on the right side of the neck and 5 cases on the left side of the neck. A total of 58 enlarged metastatic lymph nodes were included in the study, with 19 nodes in the left neck (32.76%) and 39 nodes in the right neck (67.24%). The number of lymph node metastases per person ranged from 1 to 3, with the quantities in each lymph node region (II, III, IV, V, VI) being 4, 15, 24, 4, and 11, respectively.

**Table 1:** Characteristics of the study population and cervical metastatic lymph nodes.

Variables	Group A (diameter ≤ 10mm)	Group B (10<;diameter ≤ 20)	Group C (diameter>20mm)	*P* value
Age	42.00 ± 3.26	42.40 ± 7.66	43.11 ± 7.97	0.64
Sex(male/female)	1/9	3/11	1/7	0.72
Primary tumor location (R/L)	7/3	9/5	2/5	0.19
No. of metastatic LN	19	25	14	—
Location of metastatic LN (%, R/L)				
II	0 (0)	3 (12.00,3/0)	1 (7.14,0/1)	—
III	5 (26.32,4/1)	6 (24.00,4/2)	4 (28.57,2/2)	—
IV	9 (47.37,6/3)	9 (36.00,7/2)	6 (42.86,2/4)	—
V	1 (5.26,1/0)	2 (8.00,2/0)	1 (7.14,0/1)	—
VI	4 (21.05,3/1)	5 (20.00,4/1)	2 (14.29,1/1)	—

R, right; L, left; LN, lymph node. “—” indicates that the data is not applicable.

### Treatment response of WMA

After confirming the CMLNs with FNAB, they were ablated. No enhancement was found on CEUS after ablation, indicating complete ablation of all CMLNs. **Table 2** shows that at the last follow-up, the mean largest diameter of CMLNs decreased from 7.76 ± 1.03 mm to 0.68 ± 1.66 mm (Group A) after MWA (*p <*0.05), from 13.33 ± 3.96 mm to 2.01 ± 4.54 mm (Group B) (*p <*0.05), and from 22.53 ± 5.69 mm to 5.67 ± 6.84 mm (Group C) (*p <*0.05); the mean volume decreased from 137.09 ± 55.50 mm^3^ to 8.00 ± 33.27 mm^3^ (Group A) (*p <*0.05), from 665.03 ± 536.72 mm^3^ to 93.63 ± 323.60 mm^3^ (Group B) (*p <*0.05), and from 1712.03 ± 1565.33 mm^3^ to 339.31 ± 567.31 mm^3^ (Group C) (*p <*0.05). Our results suggest that WMA is effective in reducing the diameter and volume of CMLNs. Compared with pre-ablation, which is shown in **Table 3**, the mean VRR% was 95.43 ± 19.49% (group A), 87.93 ± 34.36% (group B) and 81.44 ± 36.79% (group C), respectively. Our results showed that smaller diameter (Group A and Group B) lymph nodes, especially Group A, showed better volume reduction than the larger diameter CMLNs nodes.

**Table 2:** Changes in cervical metastatic lymph nodes and sTg level post-ablation.

Variables	Before MWA	After MWA	*P* - value
Mean largest diameter (mm)			
Group A (diameter ≤ 10mm)	7.76 ± 1.03	0.68 ± 1.66	<0.0001
Group B (10<;diameter ≤ 20)	13.33 ± 3.96	2.01 ± 4.54	<0.0001
Group C (diameter >20mm)	22.53 ± 5.69	5.67 ± 6.84	<0.0001
Mean volume (mm^3^)			
Group A (diameter ≤ 10mm)	137.09 ± 55.50	8.00 ± 33.27	<0.0001
Group B (10<;diameter ≤ 20)	665.03 ± 536.72	93.63 ± 323.60	<0.0001
Group C (diameter >20mm)	1712.03 ± 1565.33	339.31 ± 567.31	<0.01
sTg (ng/mL)			
Group A (diameter ≤ 10mm)	5.69 ± 1.65	1.20 ± 0.66	<0.0001
Group B (10<;diameter ≤ 20)	7.44 ± 2.53	1.16 ± 0.53	<0.0001
Group C (diameter >20mm)	7.78 ± 2.02	1.79 ± 0.74	<0.0001

sTg serum thyroglobulin; MWA microwave ablation; p-value of less than 0.05 was considered statistically significant.

**Table 3:** Changes in the VRR and complete disappearance rate after MWA of CMLNs.

Variables	Group A (diameter ≤ 10mm)	Group B (10<;diameter ≤ 20)	Group C (diameter>20mm)	*P* value
Mean VRR (%)	95.43 ± 19.49	87.93 ± 34.36	81.44 ± 36.79	0.28
Complete disappearance rate (%)	12/19 (63.16)	20/25 (80)	7/15 (46.67)	0.47

VRR, volume reduction ratio; MWA, microwave ablation; CMLNs, cervical metastatic lymph nodes; p-value of less than 0.05 was considered statistically significant.

After ablation, during the subsequent ultrasound follow-up, a total of 31 lymph nodes disappeared at 6 months, and a total of 39 lymph nodes disappeared at 12 months. In addition, 6 patients were found to have new CMLNs during follow-up after the initial ablation, which were subsequently ablated using MWA. No enhancement was seen on CEUS, and they disappeared at the 12-month follow-up. The complete disappearance rate of lymph nodes among the three groups were 12/19 (63.16%), 20/25(80%) and 7/15(46.67%), respectively, which is shown in **Table 3**. This suggests that lymph nodes in the 11-20 mm diameter range are more likely to disappear completely after MWA treatment, compared to smaller or larger lymph nodes. In terms of long-term efficacy, the tumor recurrence rate was Group C (2 cases, 14.29%) > Group B (2 cases, 8%)> Group A (1 case, 5.3%).

As shown in **Table 2**, compared to before ablation, the average levels of Serologic thyroglobulin (sTg) of the Group A, B and C at the last follow-up decreased from 5.69 ± 1.65 (ng/mL) to 1.20 ± 0.66 (ng/mL) (*p <*0.05), from 7.44 ± 2.53 (ng/mL) to 1.16 ± 0.53 (ng/mL) (*p <*0.05), and from 7.78 ± 2.02 (ng/mL) to 1.79 ± 0.74 (ng/mL) (*p <*0.05). As an indicator for monitoring the treatment efficacy and recurrence of thyroid cancer, the level of sTg will significantly decrease after microwave ablation. Furthermore, larger lymph nodes may face a higher risk of disease recurrence.

### Complications

After MWA, no serious complications such as bleeding or local hematoma occurred. Six patients (Group A/Group B/Group C: 3/2/1) experienced mild pain and/or burning sensation, while three patients (Group A/Group B/Group C:1/0/2) experienced transient hoarseness, which was verified by fiberoptic laryngoscopy after MWA. Therefore, the total number of complications in the three groups in this study was 4 cases (Group A), 2 cases (Group B), and 3 cases (Group C) respectively. All discomfort disappeared within one month. No local infection, cervical hematoma, skin burns, or injury to the trachea, vagus nerve, or esophagus were observed.

## Discussion

Given the difficulty of reoperation, minimally invasive thermal ablation techniques can locally control small lesions and become a new method for treating method (19, 20). In recent years, there has been an increasing amount of research on thermal ablation in the treatment of CMLNs of thyroid cancer, mostly including CMLNs between 10-20mm. Subgroup analyses are mostly based on the type of thermal ablation, such as MWA, radiofrequency ablation, and laser ablation (21–23). Additionally, current studies indicate that MWA for treating LNM is an effective and safe strategy (24, 25). Therefore, this article will, for the first time, group based on the diameter of metastatic lymph nodes to explore the effectiveness and safety of MWA of LNs.

MWA for CMLN can be used as a safe and effective alternative treatment for high-risk patients or those who refuse surgery. Our research has found that MWA is an effective method for treating cervical lymph node metastasis of thyroid cancer, with a low incidence of complications. When using the mean largest diameter and volume of MLN as grouping criteria, it was found that, compared to before surgery, the postoperative mean largest diameter and volume in the three groups were significantly reduced compared to before ablation (as shown in **Table 2**). The volume reduction rate (VRR) is commonly used as an indicator of ablation treatment efficacy, with treatment success defined as a reduction in tumor volume of over 50% (26, 27). It was found after the final follow-up that the VRR of groups A and B was greater than that of group C. CEUS examination at the final follow-up revealed that the complete disappearance rate of enlarged lymph nodes after ablation was higher in the first two groups (Group B > Group A) compared to the C group, with those not cleared showing small scar formation. And the tumor recurrence rate was Group C > Group B> Group A. Additionally, this study shows that sTg levels have significant predictive value in predicting disease metastasis and recurrence in patients with PTC (28, 29). However, due to difficulties in collecting complete data, TgAb can only be included in future studies to jointly evaluate tumor recurrence. From **Table 2**, it can be seen that there were statistically significant (*p <*0.05) differences in the pre- and post-ablation variables for all three groups. Therefore, it can be concluded that MWA is an effective method for treating PTC-CMLNs. Regarding the safety of ablation treatment, the overall complication rates for the three groups were 4/10, 2/25, and 3/14, respectively. The primary complications included mild pain and/or a burning sensation, as well as temporary voice changes. This indicates that while ablation therapy might introduce some side effects, these effects are generally mild and mostly temporary in nature. Overall, the safety of ablation therapy is relatively high, with a low incidence rate of complications, and the main complications are comparatively mild, having minimal long-term impact on patients’ health. Therefore, for patients with appropriate indications, ablation therapy can be considered a safe and effective treatment option.

MWA, as an invasive therapeutic technique, shows a certain relationship between its effectiveness and the size of the lesion (30, 31). Our research and previous studies (9, 15, 23) indicate that for smaller enlarged lymph nodes, microwave ablation can achieve good therapeutic effects. This is because their regular morphology and uniform heat conduction are conducive to complete thermal coagulation and reduced damage to surrounding tissues. However, the effectiveness of microwave ablation may be limited for larger diameter lymph node metastases, as their irregular shapes and complex internal structures, along with uneven heat conduction, may result in residual active tumor cells and complications. Additionally, for patients with larger diameter lymph nodes, after thermal ablation, the tumor size can decrease, which helps control tumor growth, alleviate patient symptoms and discomfort, increase the feasibility of surgery or radiotherapy/chemotherapy, and protect organ function. Therefore, it is important to note that although smaller lymph nodes have better treatment results, this does not mean that all patients with larger lymph nodes cannot benefit from microwave ablation. Microwave ablation remains a viable treatment option for patients whose surgical risks are too high or for whom other treatments are ineffective. Therefore, when selecting microwave ablation for the treatment of enlarged lymph nodes, it can be an effective treatment method that benefits patients, whether they have smaller or larger diameters. For patients with larger diameters who wish to achieve better therapeutic effects, it may be necessary to combine other treatment methods, such as surgical resection, chemotherapy, or radiotherapy, to achieve optimal results (32).

There are some limitations in this study. Firstly, it is a retrospective study with a relatively small sample size. Potential biases in comparing the efficacy and safety among the three groups of patients may have affected the objectivity and generalizability of the research results. Furthermore, this study focuses on the treatment of cancer with a follow-up period of only 1 year, lacking long-term follow-up observations on treatment outcomes. This limitation may have affected the comprehensive assessment of the treatment efficacy. Therefore, conclusions should be drawn with caution. Lastly, therefore, prospective, large-sample, and long-term follow-up studies are needed for future research.

In conclusion, microwave ablation (MWA) emerges as a safe and effective treatment for cervical metastatic lymph nodes from papillary carcinoma. Our study, supported by a 12-month follow-up, underscores the significant short-term benefits of managing localized lymph nodes affected by oncological diseases. Although MWA demonstrates advantages in treating smaller localized lymph nodes, its efficacy may diminish for larger diameters. Therefore, cautious consideration is advised in practical applications, and personalized treatment plans should be tailored based on a thorough evaluation of additional evidence.

## Data availability statement

The raw data supporting the conclusions of this article will be made available by the authors, without undue reservation.

## Ethics statement

The studies involving humans were approved by The First Hospital of Hunan University of Chinese Medicine. The studies were conducted in accordance with the local legislation and institutional requirements. The participants provided their written informed consent to participate in this study. Written informed consent was obtained from the individual(s) for the publication of any potentially identifiable images or data included in this article.

## Author contributions

XX: Writing – review & editing, Conceptualization, Investigation, Software, Writing – original draft. XC: Validation, Writing – original draft. JW: Writing – review & editing. PL: Writing – original draft, Data curation, Formal analysis, Investigation, Methodology, Project administration. YZ: Writing – original draft, Software, Visualization.
